# Population Geography and Emergency Contraception Access in Louisiana

**DOI:** 10.3390/pharmacy8040224

**Published:** 2020-11-20

**Authors:** Elvira Chiccarelli, Nikka Khorsandi, S. Amanda Dumas, James K. Aden, Ryan H. Pasternak

**Affiliations:** 1 Department of Pediatrics, Louisiana State University Health Sciences Center, New Orleans, LA 70118, USA; sdumas@lsuhsc.edu (S.A.D.); rpaste@lsuhsc.edu (R.H.P.); 2 School of Medicine, Louisiana State University Health Sciences Center, New Orleans, LA 70112, USA; nkhors@lsuhsc.edu; 3 Brooke Army Medical Center, US Army GME SAUSHEC, Fort Sam Houston, TX 78234, USA; james.k.aden2.civ@mail.mil

**Keywords:** emergency contraception, adolescent medicine, population geography, teen pregnancy

## Abstract

We assessed the same-day availability of oral emergency contraception (EC) in five Louisiana communities, and evaluated this data for relationships between availability and local population demographics. Researchers called all retail pharmacies in five municipalities of varying sizes in order to inquire about the same-day stockage of EC products and their availability to teens. Individual pharmacies were then geolocated to a census tract, and call data was analyzed against neighborhood census data regarding population size, income, gender, race, family structure, and educational level. A multivariable logistic regression model was performed to predict the same-day availability of emergency contraception. EC was available on the same day in 66% of all pharmacies. The same-day availability of EC decreased with the local population size (*p* < 0.001), and the availability increased with higher levels of educational attainment (*p* = 0.0015). The largest census level predictor of access to same-day EC was the city population, with the availability increasing by 6.6% for every 10,000 person increase in population. Despite changing to over-the-counter sales in 2013, EC is still not widely available in all geographic areas. Its availability is partially predictable by local population demographics, and this difference may represent a health disparity for teens and women seeking EC.

## 1. Introduction

Women can use emergency contraception (EC) after unprotected sexual intercourse to prevent an unintended pregnancy. EC options in the U.S. include: the copper intrauterine device, which must be inserted by a trained medical professional; oral ulipristal acetate (UPA), which requires a prescription; and oral levonorgestrel (LNG), which is available without a prescription [1]. All EC methods are available to teens, who tend to rely on less effective contraceptive methods, such as condoms and withdrawal [2]. When US teens do conceive, the pregnancy is unplanned in 77% of cases [3]. Therefore, in 2013 the U.S. Food and Drug Administration approved the sale of LNG to anyone regardless of age [4]. In addition, multiple professional organizations advocate for EC use for women of all ages [5,6]. For example, the American Academy of Pediatrics’ policy on EC states that physicians should provide an appropriate method of EC for teenagers in immediate need, as well as in advance of need [6]. As a result, teens’ EC usage continues to increase, and its availability has been associated with lower teen birth rates [7,8]. Despite overall national declines, there are still many areas with disproportionately high teen birth rates, including Louisiana [9]. The current literature on EC availability mainly focuses on what factors individual adolescents face when trying to access EC [10,11,12,13]. However, studying EC availability at the population level identifies possible broader-based interventions in order to improve access. Our study initially arose from a need for a local EC database for providers to counsel patients regarding where they could most likely access EC. It is important that anyone providing care for women and families at risk of an unintended pregnancy be aware of what EC availability trends might exist in their area. This research presents a rough estimate or a starting point to investigate local trends from our dataset, which encompasses a range of different-sized municipalities in Louisiana.

More than half of Louisiana women at risk of an unintended pregnancy use either an ineffective or no form of contraception [10]. Geography influences women’s access to EC, and studies have shown that women in less populous areas have both a decreased accessibility to EC and decreased EC availability in independently owned pharmacies compared to chain stores [14,15]. Additionally, women receive more misinformation about EC from pharmacies located in low-income areas [14]. Louisiana is rural and has the second highest level of income inequality in the U.S. [16]. To further understand how these geographic characteristics may influence EC access for teens, we examined how a pharmacy’s location and surrounding population could influence the accessibility of EC for adolescent patients and their physicians across Louisiana.

## 2. Materials and Methods

We obtained listings of pharmacies via Google Business for five municipalities across southern and eastern Louisiana: New Orleans, Baton Rouge, Lafayette, Monroe, and Houma. These represent major economic areas in their respective parishes (counties are called “parishes” in Louisiana) and a spectrum of population sizes from 32,864 to 343,829 in 2017 [17]. For analysis purposes, we included the area of a municipality adjacent to Monroe, called West Monroe, since it represents a contiguous area. These cities also represent the major municipalities in the catchment area of Children’s Hospital of New Orleans, the largest pediatric hospital in the state. Pharmacies were excluded if they were no longer in business, were self-identified as delivery only, or provided special order medications only when contacted. The final list used for the study consisted of 182 pharmacies. This study was formally reviewed and waivered as nonhuman subjects research by the Louisiana State University Health Science Center of New Orleans Institutional Review Board.

Researchers called each of the 182 pharmacies twice: one call was placed by a female researcher, and the other was placed by a male researcher. Researchers were medical students and pediatric residents. For each pharmacy called, researchers would either both pose as adolescent callers or both pose as physicians calling on behalf of adolescent patients. This ‘secret shopper’ methodology is a widely utilized observational research methodology in market research as well as in previous EC availability studies both before and after the change to an over-the-counter status of LNG [13,14,16,18]. Information was gathered incrementally, with female calls being made between July 2018 and January 2019 and male calls being made between October 2018 and November 2019. In the sample, there were six pharmacies that were contacted by one gender caller but not the other due to them closing in between calls. Five of these were in New Orleans, and one was in Baton Rouge; these calls were excluded from the analysis.

Data was gathered from pharmacy staff using a scripted interaction dialogue to simulate real world calls and elicit specific information. These methods and dialogue were modeled after a study series by Wilkinson et al. and used with permission [16,18]. Data gathered regarding EC included: the current same-day availability of oral EC; price; availability to a 17-year-old; and the need for parental consent for a teen purchaser. Researchers also asked the responding pharmacy staff to identify whether they were a pharmacist or not. If at any point the caller was transferred to a pharmacist, those answers and information were the ones recorded. The call length, including the hold time, was also recorded. If no pharmacy staff were available, researchers called back on another day. Findings were not validated with in-person pharmacy visits. However, male and female caller data were compared to assess concordance. The demographics and pharmacy characteristics of each study city are presented in Table 1.

Pharmacies were geolocated to a census tract, and call data was analyzed against neighborhood census data to illustrate the population represented in the area surrounding the pharmacy. The projected census data for 2017 was obtained from Geolytics [19] in order to assess for relationships between the same-day EC availability within a census tract and the following demographic characteristics: median household income, gender, households with incomes below the federal poverty level for a four-person household, households with young children under 10, race, percent married, and percent with at least a high school diploma. The estimates from 2017 were the most accurate and recent available data with corresponding demographic characteristics at the time of analysis. We purposely chose two income metrics. The median household income reflects the 50th percentile of the household income for each census tract. The percentage of households having an income under the federal poverty level for a family of four reflects the proportion of households in each locale eligible for certain federal benefits and programs [20,21].

We compared the same-day EC availability in retail pharmacies to the city population using a chi square model. The availability was compared to census variables within that block group using the Kruskal–Wallis Test. A statistical significance was established for *p*-values under 0.05. All analyses were performed using JMP v13.2 SAS Corp (Cary, NC, USA) [22]. A multivariable logistic regression model was performed to predict the same-day availability of EC. Factors significantly associated with the outcomes, including the census variables, call variables, and city population, were chosen to be in the initial model. The least significant factors were then removed through backward elimination until only factors with a *p*-value under 0.05 were left in the final model (Table 2).

## 3. Results

Overall, EC was available in 66% of all the contacted pharmacies, though only 4% of the total pharmacies carried a same-day stock of UPA. The same-day availability of emergency contraception decreased as the city population decreased, with a strong demonstrated relationship (*p* < 0.001). Additionally, there was an increasing same-day availability in census tracts with higher percentages of the population being under 10 years old (*p* = 0.0021) and higher percentages of the population having obtained a high school diploma (*p* = 0.0015). There was no apparent statistical relationship between the same-day EC availability and median household income, percent male population, percent married, percent of minority race or ethnicity, or percentage of households living below the poverty level for a family of four.

Based on a logistic regression analysis, the largest census level predictor of access to same-day EC was the city population, with the odds of availability increasing by 6.6% for every 10,000 person increase in the local population. The only other predictor of availability in this model was the percentage of the population with a high school diploma, with a 3.7% increase in the availability for every 1% increase in the rate of high school diplomates in a census tract. No other study variables remained significant in predicting the same-day EC availability using this model. The data is presented in Table 2 as reciprocal unit odds ratios, along with their corresponding 95% confidence intervals.

## 4. Discussion

This study quantified differences in the same-day EC availability across a range of different municipalities within Louisiana. Variations in the same-day availability were partially predicted by the local area’s population and educational attainment. To quantify the availability, we utilized a similar secret shopper method to what had been utilized in previous studies [13,14,16,18]. While certainly limited, this method is a useful and efficient way to estimate the prevalence of EC stockage in each geographic locale. This information can help practitioners to be aware of what patients in their local area have access to and can thus inform medical practice. Most significantly, practitioners may look to their local population size as a possible gauge of availability, as the strongest census level predictor of access to same-day EC was the city population. More research is warranted to assess whether this trend exists outside of Louisiana, but the currently available studies on rural EC access suggest that it does [13,14,15,23].

Limitations in our model include the fact that the city selection was restricted to a region of Louisiana that may not be generalizable elsewhere. We also only surveyed larger towns, rather than looking at all pharmacies across a particular area, so rural pharmacies were underrepresented. However, we presented pharmacy data from smaller locales than those from other similar studies, which does inform the discussion of EC availability. We also recognize that census data and physical location do not always align with an individual pharmacy’s customer base—some women who utilize emergency contraception likely go to a pharmacy outside of their neighborhood. Additionally, information provided over the phone may not reflect what might happen in the store, and we were unable to validate our findings in person. Some pharmacies also closed or changed ownership during the study period; we removed those from our final analysis, but this represents a change in the market that occurred during the data collection. Finally, there is an increasing presence of mail-order and online pharmacy businesses that provide EC, which our model excluded. This model is also unable to evaluate the reasons why a pharmacy may be out of stock linked to high demand or the personal and religious considerations of pharmacy owners. We were also unable to measure the effects of availability on specific patients or on any other time than when the call was placed.

Despite its limitations, this model accounted for several confounders to availability. By excluding pharmacies that self-identified as special order only, our sample was a closer representation of pharmacies where an adolescent might consider seeking EC. We also attempted to eliminate any pharmacy staff bias towards the caller and found no difference in the reported availability based on the gender or role of the caller (as an adolescent versus a physician). While our selection of cities was nonrandom, our sample did include a large range in municipality sizes.

The same-day availability of EC is an important measure of EC access, especially for adolescents who may have limited resources. The effective use of EC is time-sensitive—the earlier these medications are taken, the more likely they are to prevent an unplanned pregnancy [1]. Therefore, if a woman has trouble finding the medication or has to return to that pharmacy after the medication was ordered, the time delay represents a significant decrease in efficacy. Compared to similar studies, we found that LNG availability was lower than previously documented in other cities and in the years since the access laws changed regarding age and the over-the-counter (OTC) status [18]. There are a limited number of studies that have assessed the changes in EC availability and use since these restrictions changed. Similar studies done before the OTC status showed a significantly lower availability [16]. Studies both before and after these changes did find that rural pharmacies were less likely to stock EC [11,15,23]. Our data regarding access to UPA as an option for EC at a rate of 4% approximates the data that was previously documented, which ranged from 2.5–10% [23,24]. No pharmacies that stocked UPA lacked LNG.

We found no significant statistical relationship between income and EC availability. However, the relationship between the median household income and EC availability was close to significant (*p* = 0.0679). Using a similar model, Wilkinson et al.’s 2017 study of five large metropolitan areas correlated a decreased EC access to low-income neighborhoods [18]. Our study suggests that this trend may extend outside of urban areas to low-income census tracts in smaller communities, but a larger dataset may bring the value closer to significance. Alternatively, our study suggests that this trend may exist in low-income districts across communities of different population sizes, not just urban areas. Differences in significant findings may reflect geographic variations or a smaller sample size.

A lower EC availability was strongly correlated to lower high school graduation rates. While national high school graduation rates have improved consistently since 2010, low-income and racial minority students are still more likely to drop out of high school than their peers [25,26]. However, we did not find a statistically significant relationship between EC availability and a minority status. The poor educational attainment in EC-deprived areas may partially be the result of lower incomes, although this relationship is relatively weaker.

Other socioeconomic factors we assessed have not been previously correlated with EC availability; much of the current literature focuses on individual survey data on adolescents’ knowledge of EC and pharmacy practices [12,13,15]. However, by better quantifying the communities currently lacking EC availability, it may be easier to target improvements in EC availability as a means of reducing unplanned teen pregnancy. Other factors affecting EC utilization include patient knowledge, outpatient and emergency department clinical practices, and economic pressures for local pharmacies. Regarding teens’ use, the federal Youth Risk Behavior Survey neglects to specifically ask questions on EC use and only addresses other forms of contraception [2]. Overall, there is still a lot to learn about how best to address the barriers teens face in accessing EC, and investigations are needed to further explore the role geographic factors play in reproductive health disparities.

## Figures and Tables

**Table 1:** Demographics and pharmacy characteristics of the study cities.

Louisiana City	Total Population ^a^	Same-Day LNGAvailability (%) ^b^	Retail Pharmacy Density (per 10,000 People) ^c^	Teen Birth Rate ^d^	Nonwhite Population (%) ^a^	Households below Federal Poverty Level (%) ^a^	Population with Less than High School Education (%) ^a^
New Orleans	343,829	88	0.75	18.1	65.7	15.4	21.3
Baton Rouge	229,493	68	0.59	12.9	61.6	13.7	19
Lafayette	127,657	54	0.37	24.0	34.8	9.3	17.3
Monroe	61,880	56	0.19	14.8	58.2	16.8	22.4
Houma	32,864	52	0.13	17.5	29.2	11.7	27.4

^a^ U.S. Census data obtained through Geolytics for 2017; ^b^ Pharmacy calls in city reporting same-day oral levonorgestrel stock; ^c^ Total retail pharmacies per city, July 2018; ^d^ Reported as births per 1000 females aged 15–19 years in that city’s parish, 2017.

**Table 2:** Multivariate logistic regression for the same-day emergency contraception availability at selected Louisiana pharmacies.

Predictor	β	SE β	Wald’s γ^2^	*p*	Reciprocal Odds Ratio, per Unit Change in Regressor	95% Confidence Interval
City Population/10,000	0.0064	0.0013	27.0308	<0.001	1.066	0.914–0.962
Population with High School or Higher Education (%)	3.6360	1.1369	10.4805	0.00121	1.037	0.943–0.986
